# Supplementary material for: Individual retrotransposon integrants are differentially controlled by KZFP/KAP1-dependent histone methylation, DNA methylation and TET-mediated hydroxymethylation in naïve embryonic stem cells
Source: Epigenetics Chromatin. 2018 Feb 26;11:7. doi: 10.1186/s13072-018-0177-1 (PMC6389204; doi:10.1186/s13072-018-0177-1)
Supplement: Supplementary file 11 — Additional file 11. Pattern analysis. [file 13072_2018_177_MOESM11_ESM.zip › Patterns analysis/DataTables/extensions/Responsive/examples/child-rows/index.html]

Responsive examples - Child row control


# Responsive example Child row control

When a column is removed from display by Responsive, the data is still available in the table and
can be displayed in a DataTables *child row* (see `row().child()DT`). By default Responsive will show child
row controls in the first column when the table has been collapsed, allowing the end user to show /
hide the information from the hidden columns.

Responsive has a number of options for display of the child rows:

- If child row display is enabled: `responsive.detailsR`
- How the show / hide control is displayed: `responsive.details.typeR`
- How the child row is rendered: `responsive.details.rendererR`

This section shows examples of these options being used.

### Child rows

- Disable child rows
- Column controlled child rows
- Column control - right
- Whole row child row control
- Custom child row renderer

Please refer to the DataTables documentation for full
information about its API properties and methods.  
Additionally, there are a wide range of extras and
plug-ins which extend the capabilities of
DataTables.

DataTables designed and created by SpryMedia Ltd © 2007-2014  
DataTables is licensed under the MIT license.
